# Case report: A rare case of cerebral herniation during glioma resection in a syphilis-positive patient

**DOI:** 10.3389/fneur.2023.1196431

**Published:** 2023-08-10

**Authors:** Han Wang, Qianli Lin, Fang Wang, Yong Yi, Xiaoping Xu, Jingcheng Jiang, Qingshan Deng

**Affiliations:** ^1^Department of Neurosurgery, The Second People's Hospital of Yibin, Yibin, Sichuan, China; ^2^Department of Clinical Lab, The Second People's Hospital of Yibin, Yibin, Sichuan, China

**Keywords:** cerebral herniation, glioma, syphilis, vasculitis, craniotomy

## Abstract

Acute intraoperative cerebral herniation is catastrophic in craniotomy and seriously affects the outcomes of surgery and the prognosis of the patient. Although the probability of its occurrence is low, it can lead to severe disability and high mortality. We describe a rare case of intraoperative cerebral herniation that occurred in a syphilis-positive patient. The patient was diagnosed with both glioma and syphilis. When the glioma was completely removed under the surgical microscope, acute cerebral herniation occurred. An urgent intervention in cerebral herniation identified a collection of colorless, transparent, and protein-rich gelatinous substances rather than a hematoma, which is a more commonly reported cause of intraoperative cerebral herniation in the literature. We have found no previous descriptions of such cerebral herniation during craniotomy in a patient with syphilis and glioma. We suspected that the occurrence of intraoperative cerebral hernia might be related to the patient’s infection with syphilis. We considered the likelihood of an intraoperative cerebral herniation to be elevated when a patient had a disease similar to syphilis that could cause increased vascular permeability.

## Introduction

Acute intraoperative cerebral herniation is more common in brain trauma surgery but can occasionally occur in surgery for brain tumor resection. Based on prior literature search, the etiology of intraoperative acute intraoperative brain bulge encompasses the occurrence of hematoma in a distant location from the surgical site, acute cerebral edema resulting from obstruction of cerebral venous return during surgery in the patient (1). It seriously affects the prognosis of patients and does more harm than the disease itself. To the best of our knowledge, there are no previous descriptions of an acute intraoperative epidural lesions in a glioma patient with syphilis that resulted in acute cerebral herniation.

## Case presentation

### Clinical presentation

A 28-year-old woman was admitted to the hospital for mental disorders half a month prior. Severe headaches and visual hallucinations were the primary clinical manifestations without fever, heart murmur, skin lesionss, or hepatosplenomegaly. Motor and sensory examinations revealed normal results. The bilateral Babinski sign was negative.

### Diagnosis and preoperative course

**Figure 1:**
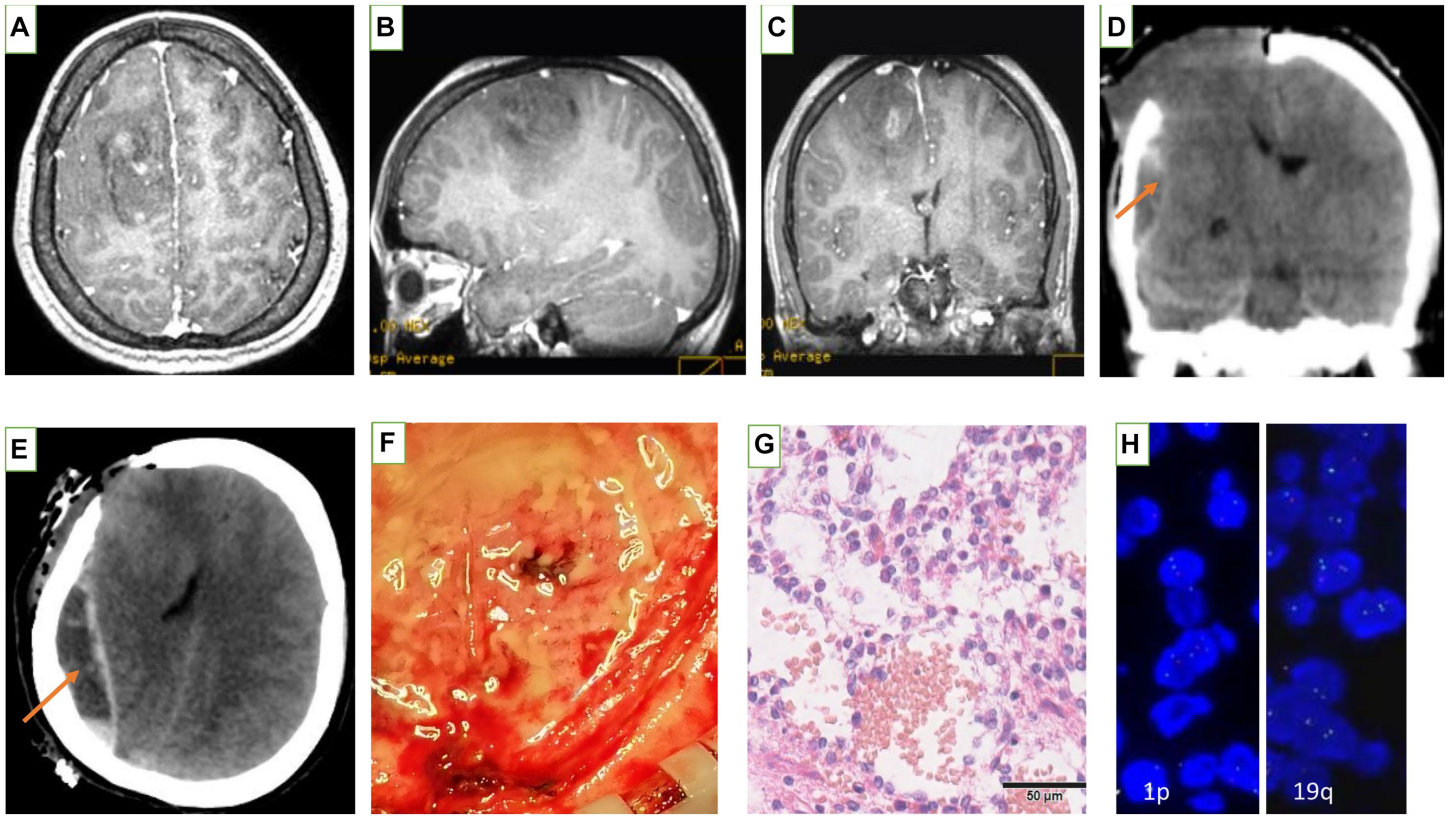
Images obtained from the preoperative cranial MRI: the right frontal lobe space-occupying lesions. **(A)** axial T1 enhanced, **(B)** Sagittal T1 enhanced, and **(C)** Coronal T1 enhanced. **(D,E)** Emergency cranial CT during the operation: found the low-density of the epidural behind the surgical area. (orange arrow). **(F)** Photo of the epidural during decompressive craniectomy: gelatinous substance in the epidural area. **(G,H)** Pathological examination confirm the right frontal lobe mass is anaplastic oligodendroglioma, IDH mutated, 1p/19q codeleted, (WHOIII): scale bar 50um.

Magnetic resonance imaging (MRI) of the head showed a space-occupying lesions in the right frontal lobe (Figures 1A–C). The toluidine red unheated serum test (TRUST) titer was 1:8. The *Treponema pallidum* particle agglutination test (TPPA) was 1:80 positive, and serology was negative for human immunodeficiency virus (HIV). We could not perform a preoperative CSF examination on this patient, as we considered that she had features of increased intracranial pressure. At the same time, the patient was unable to cooperate with the lumbar puncture due to her psychiatric symptoms. She received penicillin G intravenously at a dosage of 2.4 million units per day for 14 days in order to rule out cerebral syphilis gumma (2). Mannitol is simultaneously infused intravenously to reduce her intracranial pressure. A follow-up computed tomography (CT) scan of the head after treatment showed that the lesions was almost unchanged from the time of admission.

### Operation and pathological findings

A craniotomy was performed after signing the consent form.We used a large craniotomy with a bone flap that exceeded the extent of the patient’s tumor edema. The operation went well, and the tumor was basically resected. When we were preparing to suture the dura mater, the patient suddenly developed an acute protrusion of the right frontal lobe within approximately 10 min. An emergency CT scan showed a low-density, spindle-shaped epidural lesions (Figures 1D,E). Decompression craniectomy and evacuation of the epidural lesionss were performed immediately. A large amount of colorless and transparent gelatinous substances were found in the epidural area of the patient (Figure 1F). It was easily evacuated by using a surgical aspirator. After the operation, the patient was in a coma for 3 days and gradually became conscious but was accompanied by left hemiplegia. The biochemical examination of exudate epidural fluid contains a large amount of proteins. The cytological examination of exudate epidural fluid contains a small amount of red blood cells. After the patient became conscious, her headache and the mental disorder of the patient were relieved. As soon as the patient’s consent was obtained, we performed a lumbar puncture 2 weeks after her surgery. Cerebrospinal fluid (CSF) analysis revealed a white blood cell count of 20.2 × 10^6^/L (85% lymphocytes and 15% monocytes), a red blood cell count of 1.03 × 10^9^/L, a total protein level of 0.79 g/L, and a chloride concentration of 122.6 mmol/L. Her CSF analysis revealed that TRUST was negative and TPPA was 1:40 positive. Pathological examination showed H&E staining of a pleomorphic glial tumor with round tumor cells and high mitotic activity (Figure 1G). Immunohistochemistry showed that glial tumor cells were positive for GFAP (glial fibrillary acidic protein). Nuclear expression of ATRX (α-thalassemia/mental retardation syndrome X-linked) was retained, and IDH1 (isocitrate dehydrogenase 1) R132H mutant protein was expressed. The Ki-67 proliferative index was 5%. Analysis of 1p and 19q status revealed a combined loss of 1p and 19q. The right frontal lesions was graded as anaplastic oligodendroglioma, IDH mutated, 1p/19q codeleted, WHO III (Figures 1G,H).

### Postoperative course

After the operation, the patient was in a coma for 3 days, gradually became conscious, and was discharged after 1 month of treatment but was accompanied by left hemiplegia. Because the patient lived in a remote village, the telephone follow-up revealed that she was able to walk with a cane. The patient currently felt headaches all the time and needed to be relieved by taking painkillers.

## Discussion

**Figure 2:**
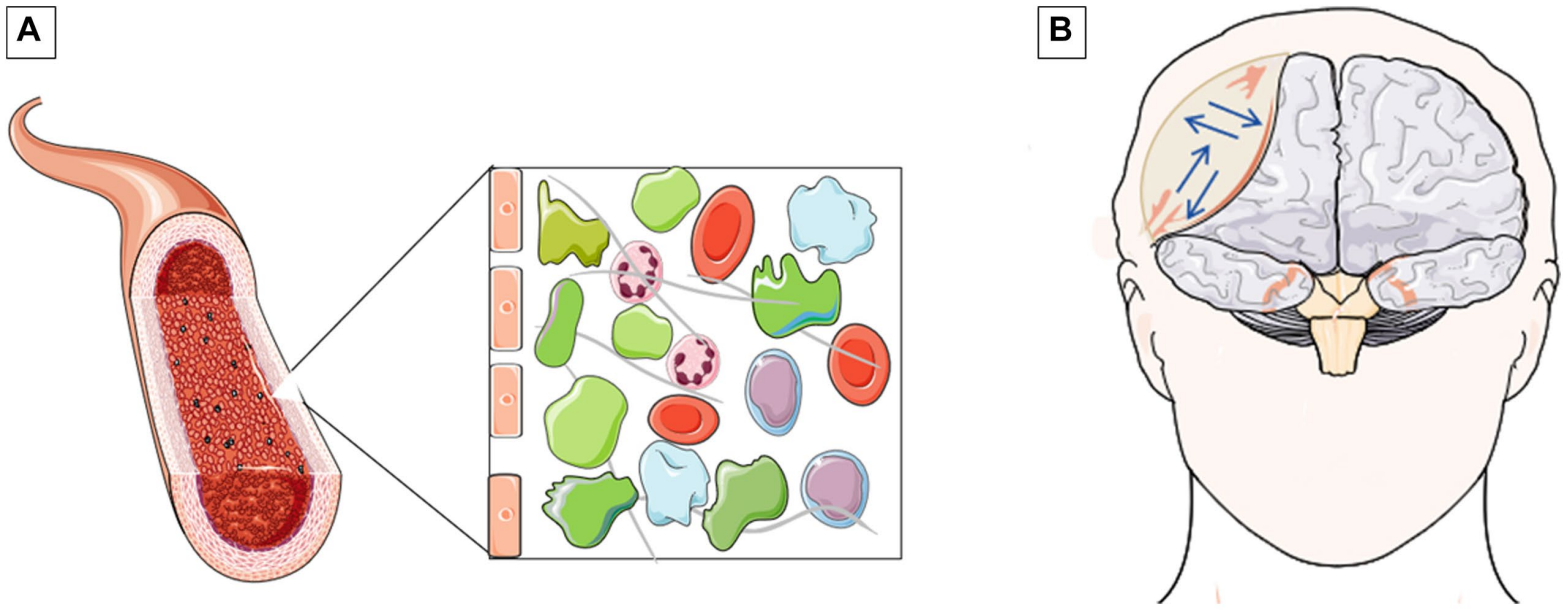
Schematic of inflammatory vascular exudation and schematic of the principle of epidural space occupying lesion formation. **(A)** Inflammation of blood vessels results in increased distance between endothelial cells. Eventually, a large amount of proteinaceous substances leak out of the blood vessels. **(B)** The exuded proteins churned in all directions with the frequency of the patient's cerebral pulsatility, eventually leading to the epidural mass effect (blue arrows).

Acute intraoperative cerebral herniation is an infrequent but catastrophic occurrence. According to previous reports, it often occurs in emergency surgery for severe craniocerebral trauma, and the common cause of intraoperative acute cerebral herniation is epidural or subdural hemorrhage during surgery (1). To date, a case of intraoperative cerebral herniation due to a large amount of colorless and transparent gelatinous substances has not been reported. Based on the patient’s symptoms, serology, and CSF findings, we considered the patient to have neurosyphilis. According to previous reports, neurosyphilis can occur at any time after primary infection (3, 4). At the same time, the pathologic findings of meningovascular syphilis include diffuse thickening and lymphocytic infiltration of the meninges with superimposed arteritis (5, 6). The formation of the epidural gelatinous substance in this patient can be attributed to the following reasons. First, the decrease in intracranial pressure after tumor resection leads to an increase in volemia of the epidural blood vessels (7). Second, patients with neurosyphilis have vasculitic changes on the dural surface and increased vascular permeability (8) (Figure 2A). Glioma can form neovascularization with high defects, resulting in vessels with abnormal morphology and function (9). Third, leakage of plasma-protein-rich fluid accumulates in the epidural space, which is stirred repeatedly as the patient’s cerebral pulsatility to form a gelatin-like substance (Figure 2B). According to the CT imaging and intraoperative images, the epidural collection is not only gelatinous but also has a blood component. Young and middle-aged postoperative patients with postoperative intracranial tumors often suffer from epidural hemorrhage because the dura mater is more loosely combined with the skull, and it is easier to peel off when the intracranial pressure changes. Therefore, epidural hemorrhage in remote sites is more likely to occur. In elderly patients, due to the large gap between the dura mater and the brain tissue and the poor compliance of the brain tissue in elderly patients, the intracranial pressure fluctuates significantly during the operation, which may easily lead to stretching and rupture of the bridging vein. Therefore, subdural hematomas in distant parts are more likely to occur (10, 11). Eventually, the gradually increasing epidural collection causes a malignant intraoperative cerebral herniation.

## Conclusion

Our case is the first known case of a malignant bulge of the brain during craniotomy caused by the accumulation of a large amount of gelatinous substances. When cerebral herniation occurred, we intervened promptly and accurately, which ultimately led to the patient’s survival. Despite the efficacy of the treatment for neurosyphilis in this particular patient, the irreversible harm inflicted by treponema pallidum on the blood vessels of the meninges necessitates caution among patients undergoing craniotomy, as cerebral bulges may arise during surgery, regardless of whether it occurs during the active phase of neurosyphilis or subsequent to treatment. In conclusion, it is strongly advised that neurosurgeons exercise utmost caution during craniotomy procedures conducted on patients presenting with treponema pallidum infection or any other medical condition that may potentially induce vasculitis. When these patients require a craniotomy, the likelihood of cerebral herniation during surgery will increase.

## Data availability statement

The original contributions presented in the study are included in the article/supplementary material, further inquiries can be directed to the corresponding author.

## Ethics statement

Ethical review and approval was not required for the study on human participants in accordance with the local legislation and institutional requirements. Written informed consent from the patients/ participants or patients/participants' legal guardian/next of kin was not required to participate in this study in accordance with the national legislation and the institutional requirements. Written informed consent was obtained from the individual for the publication of any potentially identifiable images or data included in this article.

## Author contributions

All authors listed have made a substantial, direct, and intellectual contribution to the work and approved it for publication.

## Funding

This work was supported by the Sichuan Province Key R&D Project (22ZDYF1826) and the 2021 hospital-level incubation Project of Yibin Hospital, West China Hospital of Sichuan University (2021FY15).

## Conflict of interest

The authors declare that the research was conducted in the absence of any commercial or financial relationships that could be construed as a potential conflict of interest.

## Publisher’s note

All claims expressed in this article are solely those of the authors and do not necessarily represent those of their affiliated organizations, or those of the publisher, the editors and the reviewers. Any product that may be evaluated in this article, or claim that may be made by its manufacturer, is not guaranteed or endorsed by the publisher.
